# The Texas State Medical Association

**Published:** 1902-04

**Authors:** 


### Society Notes.

### The Texas State Medical Association.

It was hoped and expected that the Journal would publish in this issue the program for the Dallas meeting (May 6-9), but to the hour of going to press it has not been received.

Easterly, Texas, March 17, 1902.

*To the Texas Medical Journal, Austin, Texas:*

The thirteenth semi-annual meeting of the Brazos Valley Medical Association will convene at Marlin, Texas, on the second Tuesday and Wednesday in May, proximo, the same being the 13th and 14th days of the month. A good program will be furnished and a full attendance is desired.

W. B. Briggs, Secretary.

Daniel Parker, President.

